# 维奈克拉联合去甲基化药物诱导治疗急性白血病患者发生肿瘤溶解综合征7例报告并文献复习

**DOI:** 10.3760/cma.j.issn.0253-2727.2023.06.013

**Published:** 2023-06

**Authors:** 虞莎 郭, 辰昊 赵, 德媛 胡, 凯 沈, 苏宁 陈

**Affiliations:** 1 苏州大学附属第一医院血液内科，苏州 215006 Department of Hematology, the First Affiliated Hospital of Soochow University, Suzhou 215006, China; 2 苏州大学附属第二医院检验科，苏州 215004 Department of Clinical Laboratory, the Second Affiliated Hospital of Soochow University, Suzhou 215004, China; 3 江苏省血液研究所，苏州 215006 Jiangsu Institute of Hematology, Suzhou 215006, China

维奈克拉联合去甲基化药物在急性白血病患者治疗过程中，可诱发以高钾、高磷、高尿酸、低钙、肾损害等为特征的肿瘤溶解综合征（TLS）。为探讨维奈克拉联合去甲基化药物用于急性白血病治疗的安全性，我们回顾性分析了本中心7例使用维奈克拉联合去甲基化药物诱导治疗的急性白血病患者发生TLS的诊疗过程、治疗前后相关实验室指标、干预措施等，并复习相关文献，报告如下。

## 病例与方法

收集苏州大学附属第一医院血液科2021年2月至2022年6月诊治的7例使用维奈克拉联合去甲基化药物诱导治疗发生TLS的急性白血病患者临床资料。回顾性分析患者初诊时临床及实验室特征、发生TLS前后的实验室检查结果、干预措施以及转归等。

## 结果

1. 临床及实验室特征：7例均为初治患者，包括急性髓系白血病6例、急性混合细胞白血病1例，男3例、女4例，中位年龄44（14～65）岁。通过治疗前脏器功能评估，未见明显肾功能不全者。其中2例合并其他基础疾病：例3既往高血压病史4年，未规律口服药物及监测血压，另因血糖升高5年余间断口服降糖药物治疗，未规律监测血糖；例6入院后影像学检查提示合并支气管扩张、脂肪肝。7例患者初诊时血常规、外周血原始细胞比例、骨髓MICM结果等临床特征见表1，诱导治疗方案以及发生TLS前后实验室检查结果见表2。在诱导治疗前，7例患者中位WBC 44.3（2.0～125.4）×10^9^/L，HGB 91（44～124）g/L，PLT 46（24～151）×10^9^/L；外周血原始细胞比例增高者5例；骨髓MICM检查中，染色体核型异常者3例，多重PCR检出常见白血病融合基因4例：CBFβ-MYH11、SET-NUP214、NUP98-KDM5A、MIL-AF9各1例；高通量检测筛查血液系统疾病相关基因突变示：WT1突变2例，FLT3-ITD（high）突变2例，KRAS突变2例，NRAS突变2例，CEBPA双等位基因、KIT、ETV6剪切变异、NPM1、BCORL、PTPN11、TP53、BCOR、IDH2突变各1例。

治疗方案上，4例患者采用维奈克拉联合阿扎胞苷方案治疗，其中2例严格遵循维奈克拉剂量爬坡给药原则，另2例维奈克拉剂量为200 mg第1～14天；3例患者采用维奈克拉联合地西他滨方案治疗，其中2例严格遵循维奈克拉剂量爬坡给药原则，另1例则因患者使用伏立康唑预防真菌感染，维奈克拉剂量调整为100 mg 第1～28天。治疗前血常规WBC>30×10^9^，即呈现高肿瘤负荷者共4例，其中位WBC 77.85（44.3～125.4）×10^9^/L，我们均予羟基脲、小剂量阿糖胞苷（Ara-C）进行降WBC预处理。其中3例呈明显下降水平：例2降至2.37×10^9^/L，例4降至25.1×10^9^/L，例5降至32.4×10^9^/L；例1外周血WBC始终仍呈持续升高趋势，升至 167.6×10^9^/L。

根据开始治疗后的临床症状及实验室指标分析，7例发生TLS的患者中3例发生于治疗第2天（+2 d），2例发生于+3 d，2例发生于+1 d；其中2例患者在开始治疗前即有部分血生化常规、电解质指标异常：例1血磷、肌酐偏高；例5血钙偏低，乳酸脱氢酶偏高。7例患者发生TLS时的血生化常规、电解质结果中，呈血钾升高者2例，呈血磷升高者5例，呈血钙降低者4例，呈肌酐升高者3例，呈尿酸升高者4例，另有乳酸脱氢酶显著升高者4例，提示可能存在短时间内大量的肿瘤细胞裂解；天冬氨酸转氨酶升高者2例，提示同时发生了肝细胞损伤。在发生TLS的同时，2例患者出现了明显的出凝血功能异常：例4于+2 d出现低血压、齿龈出血等出凝血功能异常的临床症状，查凝血功能示：凝血酶原时间20.20 s，活化部分凝血活酶时间35.20 s；D-二聚体>40 mg/L；例5于+1 d查凝血功能：部分凝血活酶时间47.2 s，凝血酶时间26.8 s，凝血酶原时间26.0 s，纤维蛋白降解物150 mg/L，纤维蛋白原0.27 g/L，D-二聚体46.52 mg/L，3P阳性（+），ISTH评分11分，提示患者发生了DIC。另外，4例患者在发生TLS的同时疑似伴有感染：例4于+2 d出现发热伴畏寒，热峰39.1 °C；例5入院胸部CT提示双肺多发磨玻璃影、双肺下叶炎症、胸腔积液，+1 d出现胸闷持续加重合并血氧饱和度进行性下降；例6治疗前结核感染T细胞检测阳性，痰液高通量病原体测序提示：副流感嗜血杆菌（序列数：31516），肠系膜明串珠菌（序列数：221），鲍曼不动杆菌（序列数：199），肺炎克雷伯菌（序列数：72），人疱疹病毒（序列数：1537），EB病毒（序列数：7）；例7于+1 d出现发热伴明显畏寒、寒战，热峰39.1 °C，并出现血压、血氧饱和度持续下降。

**表1:** 7例使用维奈克拉联合去甲基化药物诱导治疗发生肿瘤溶解综合征的急性白血病患者初诊时临床特征

例号	性别	年龄（岁）	血常规	外周血原始细胞	骨髓细胞形态学	流式细胞术免疫分型	染色体核型	多重PCR	二代测序
1	男	44	WBC 125.4×10^9^/L HGB 93 g/L PLT 24×10^9^/L	75%	AML，M_1_可能，M_5_待排除	分析93.3%幼稚细胞群，为髓系抗原表达	未见明显异常	阴性	CEBPA双等位基因、WT1基因突变阳性,FLT3-ITD(high)
2	女	20	WBC 51.5×10^9^/L HGB 124 g/L PLT 42×10^9^/L	60%	原始幼稚细胞占68%；POX染色：96%阳性；考虑AML，M_4E0_或M_2_可能	CD34、HLA-DR、CD13、CD33、CD117阳性，CD38弱阳性，为髓系表达	未见明显异常	CBFβ-MYH11(+)	KIT(p.Asp816Val) 40.4%
3	男	64	WBC 3.4×10^9^/L HGB 100 g/L PLT 151×10^9^/L	52%	增生极度活跃，原始幼稚细胞占63%, POX染色：55%阳性	分析79.5%幼稚细胞群，为髓系、T淋系混合表达	未见明显异常	SET-NUP214(+)	ETV6剪切变异49.7%
4	女	14	WBC 44.3×10^9^/L HGB 52 g/L PLT 46×10^9^/L	91%	增生明显活跃，原始粒细胞占72.8%，POX染色：70%，AML骨髓象	分析79.5%幼稚细胞群，为髓系抗原表达	未见明显异常	阴性	NPM1(p.W288fs) 46.12%, FLT3-ITD 69.69%, BCORL(p.R1183W) 52.37%
5	女	21	WBC 104.2×10^9^/L HGB 44 g/L PLT 28×10^9^/L	82%	AML骨髓象	分析91.2%幼稚细胞群，为髓系抗原表达	47,XX,+6[4]/46,XX,[11]	NUP98-KDM5A(+)	KRAS、PTPN11基因突变阳性
6	男	44	WBC 3.3×10^9^/L HGB 91 g/L PLT 47×10^9^/L	无	AML骨髓象	分析45.5%幼稚细胞群，为髓系抗原表达	复杂核型^a^	MIL-AF9(+)	NRAS、TP53、KRAS基因突变阳性
7	女	65	WBC 2.0×10^9^/L HGB 91 g/L PLT 48×10^9^/L	无	有核细胞增生明显活跃，原始幼稚细胞占78.4%，诊断AML	分析87.75%幼稚细胞群，为髓系抗原表达，符合AML-M_1_/M_2_表型，表达HLA-DR、CD13、CD33、CD117,弱表达CD45	47,XX,+4[8]/46,XX,[2]	阴性	BCOR(p.Gly974fs) 41.80%, IDH2 (p.Arg172fs) 43.30%, NRAS(p.Gly12Arg) 1.60%、(p.Gly12Asp) 38.30%、(p.Gly13Asp) 4.80%, WT1(p.His450fs) 38.90%、(p.His470Arg) 3.00%

注 ^a^核型为46,XY,+8,?dic(15;17)(q11;p11)[2]/46,idem,t(9;11)(p22;q23)[8]

2. 干预措施及转归：在7例患者的治疗过程中，我们均充分辅以水化、碱化、利尿、降尿酸、脏器保护等支持治疗，并动态监测血生化常规。在临床症状及实验室指标提示发生TLS后，我们均予暂停治疗用药，并进一步加强水化、碱化、利尿，同时纠正电解质紊乱。针对例1、例7肌酐、尿酸水平显著升高，我们考虑合并肾功能不全，进一步予连续性肾脏替代治疗（CRRT）减轻脏器负荷。针对例3肌酐升高以及高血压、血糖升高病史，我们予前列腺素E1注射液、复方α-酮酸片、尿毒清颗粒以改善循环、保护肾脏，并严密监测患者血糖及血压。对于出现明显凝血功能异常的患者（例4、例5），我们予血浆、血小板、纤维蛋白原、凝血酶原复合物输注支持治疗，纠正出凝血异常。对于怀疑伴有感染的4例患者，我们在完善病原学检查的基础上，经验性广覆盖抗感染，并密切监测其血生化常规及电解质、凝血功能、心功能、感染指标等。

例1在CRRT治疗后TLS相关指标有所好转，我们再次予以羟基脲、小剂量Ara-C降WBC处理后继续以原方案诱导治疗，患者1个疗程达完全缓解（CR），目前于我院巩固治疗。例2在TLS发生2 d后相关指标呈好转趋势，继续恢复治疗用药，1个疗程达CR，后分别予以IA方案、阿扎胞苷联合中剂量Ara-C、IA方案巩固治疗共2个疗程后行异基因造血干细胞移植，目前定期门诊随访。例3在TLS发生5 d后相关指标有所好转，提示脏器功能逐步恢复，继续原方案治疗，1个疗程达CR，目前于当地医院巩固治疗。例4在TLS发生3 d后相关指标好转，原方案1个疗程未缓解，予IA方案再诱导治疗缓解，目前于当地医院巩固治疗。例5在相关指标恢复正常后继续原方案治疗，1个疗程达CR，在予维奈克拉联合阿扎胞苷方案巩固治疗1个疗程后，患者本病复发，因凝血异常发生颅内出血、大脑镰下疝，最终自主呼吸、心律无法恢复，自动出院。例6在TLS相关指标有所好转后恢复用药，治疗结束后5 d复查骨髓示本病未缓解，复查胸腹部CT示：两肺炎症；双侧锁骨上下窝、左侧腋窝、腹膜后、肠系膜多发淋巴结肿大；胸椎椎体旁多发软组织影；脾脏肿大；腹腔积液。患者同时出现明显腹胀、腹围进行性增加，考虑白血病髓外浸润不能除外。后家属放弃治疗，患者自动出院。例7经CRRT及抗感染治疗后，感染性休克纠正，TLS相关指标有所好转。但因患者本病未得控制，血象三系持续低下，出现眼部软组织感染，加强抗感染后疗效不佳；且间断烦躁、意识障碍，查双侧病理征阳性，结合头颅CT平扫未见明显出血表现，考虑中枢神经系统感染不能除外。后家属放弃治疗，患者自动出院。

**表2:** 7例使用维奈克拉联合去甲基化药物诱导治疗的急性白血病患者发生肿瘤溶解综合征(TLS)前后生化及电解质结果、诱导治疗方案

例号	治疗前是否存在血生化、电解质异常	诱导治疗方案	发生TLS时血生化、电解质异常
1	血磷1.87 mmol/L 肌酐140.9 µmol/L	维奈克拉200 mg第1~14天，阿扎胞苷100 mg第1~4天、200 mg第5~7天	血钾5.83 mmol/L，血磷3.23 mmol/L，肌酐288 µmol/L，尿酸905 µmol/L
2	否	维奈克拉100 mg第1天、200 mg第2天、400 mg第3~28天，地西他滨30 mg第1~5天	血钙1.59 mmol/L，血磷3.2 mmol/L，尿酸1048.1 µmol/L，LDH 7 065.8 U/L
3	否	维奈克拉100 mg第1天、200 mg第2天、400 mg 第3~14天，阿扎胞苷100 mg第1~4天、200 mg第5~7天	血钙1.91 mmol/L，肌酐175 µmol/L
4	否	维奈克拉200 mg第1~14天，阿扎胞苷100 mg第1~4天、200 mg第5~7天	血钙1.80 mmol/L，血钾5.12 mmol/L
5	血钙2.05 mmol/L LDH 389.7 U/L	维奈克拉100 mg第1天、200 mg第2天、400 mg 第3~28天，地西他滨30 mg第1~5天	血磷1.93 mmol/L，血钙1.67 mmol/L，尿酸461.6 µmol/L，LDH 2 690.6 U/L
6	否	维奈克拉100 mg第1~28天，地西他滨40 mg第1~5天	尿酸 401.9 µmol/L，血磷1.66 mmol/L，AST 247.2 U/L，LDH 11 632.7 U/L
7	否	维奈克拉100 mg第1天、200 mg第2天、400 mg第3~28天，阿扎胞苷100 mg第1~9天	AST 773 U/L，LDH 64 658 U/L，血磷2.12 mmol/L

## 讨论及文献复习

维奈克拉是一种具有高选择性的BCL-2抑制剂，2020年12月2日起，我国国家药品监督管理局批准维奈克拉联合阿扎胞苷用于治疗因合并疾病而不适合接受强诱导化疗或年龄≥75岁的AML成年患者[1]。目前，也有更多的研究将该方案应用至年轻的AML患者、难治/复发的AML患者的治疗中[2]。TLS是肿瘤治疗过程中的一种常见急症。它是由于大量肿瘤细胞裂解破坏后释放其内容物，所导致的一组代谢异常、甚至可引起机体多器官损伤的症候群，最常见于治疗开始后1周左右的时间，严重时进展迅速可危及生命[3]。TLS的显著特点表现为高尿酸血症、高钾血症、高磷血症和低钙血症等电解质紊乱，这些代谢异常可进一步进展为临床毒性作用，致使肾功能衰竭、心律失常、癫痫发作甚至死亡[3]–[4]。

维奈克拉最早获批应用于慢性淋巴细胞白血病（CLL）/小淋巴细胞淋巴瘤（SLL）的治疗，在高达13.4％的CLL病例中显示出与TLS的发生高度相关[5]，这可能是维奈克拉可在给药后数小时内迅速诱导细胞凋亡和使肿瘤体积急剧减小造成的[6]。而在针对AML的治疗中，基于维奈克拉的诱导治疗方案引起的TLS则很少见，维奈克拉联合阿扎胞苷的Ⅲ期临床试验显示，TLS发生率仅为1.1％[7]。在由中国临床肿瘤学会（CSCO）白血病专家委员会发布的《维奈克拉治疗恶性血液病临床应用指导原则（2021年版）》[1]中，为降低接受维奈克拉联合方案治疗的AML患者发生TLS的风险，给出的建议包括：在首次给药前，应充分评估WBC、尿酸水平及肌酐清除率等基础指标，并预防性提供水化和抗高尿酸血症的药物；在治疗过程中还需注意：①充足水化；②治疗前通过给予羟基脲口服或白细胞单采术，将WBC降至25×10^9^/L以下（尤其是伴有NPM1或IDH突变患者，可考虑降至10×10^9^/L以下）[8]；③治疗前应评估血生化，并纠正已存在的异常情况；④注意维奈克拉的剂量爬坡给药；⑤给药前、爬坡期内密切监测血生化以评估TLS；⑥当患者合并肾功能不全时，需加强预防、更密切监测[8]–[9]；⑦由于维奈克拉在体内主要通过细胞色素P450 3A（CYP3A）代谢，在与中等或高强度CYP3A抑制剂联用时，需要调整维奈克拉的剂量。而一旦发生与TLS相关的血生化改变或临床症状时，需暂停用药。

Shahswar等[10]报道，截至2020年5月，其单中心42例复发难治或初诊AML患者在接受维奈克拉联合去甲基化药物或者低剂量阿糖胞苷后，共5例（12％）患者发生了实验室TLS，其中1例治疗前即因慢性终末期肾病而接受透析治疗的患者出现了肌酐水平显著升高，达到了临床TLS的诊断标准。研究者总结发现，基于维奈克拉的诱导治疗方案所引起的TLS患者相对更为年轻，且基本均为初诊AML，且更多地使用伏立康唑而非泊沙康唑作为抗真菌药物。Kim等[11]报道1例欧洲白血病专家组ELN预后良好组、外周血肿瘤负荷偏低、未见其他明显TLS高危因素的74岁男性AML患者，在规范的维奈克拉联合阿扎胞苷诱导治疗开始后的12 h之内发生了TLS。研究者推测合并症、体能评分、疾病负荷状态、肾脏功能均有可能是老年AML患者在该方案治疗下发生TLS的相关因素。

在我们报道的本中心7例维奈克拉联合去甲基化药物诱导治疗发生TLS的急性白血病患者中，治疗前呈高肿瘤负荷的4例患者，我们均预先进行降WBC预处理。其中例1由于外周血肿瘤负荷仍呈不断升高趋势、病情进展迅速，我们不得不在WBC未降至25×10^9^/L以下的情况下开始维奈克拉与去甲基化药物的使用。由于个体情况差异巨大，在临床实践中常难以严格遵照指南执行白细胞计数标准开展治疗，这是致使大量肿瘤细胞在短时间内裂解从而导致TLS发生的原因之一。例4～例7即便在治疗前WBC达到指南要求，由于可能存在合并感染导致机体组织细胞代谢紊乱与一定程度的微循环障碍，尤其例4、例5同时合并出凝血功能异常，可能也是TLS发生的诱因之一。例3则有多年高血压、高血糖病史，虽外院治疗前未见其存在明显肾功能不全，但不能排除该患者因基础疾病致肾脏微血管内皮细胞存在损伤，从而在用药过程中因肾功能受损而更易发生TLS。在这7例患者的治疗过程中，我们遵循维奈克拉剂量爬坡给药原则，联合使用唑类抗真菌药物时我们将维奈克拉剂量严格降低至稳定期100 mg/d[1]；且7例均予以充分静脉水化碱化、口服别嘌呤醇降尿酸等支持治疗，并动态监测血生化、电解质。当实验室结果提示出现TLS后，7例患者均予迅速暂停用药、纠正电解质紊乱，并予加强水化碱化、利尿、降尿酸；出现尿素氮、肌酐显著升高、尿量显著减少及恶心呕吐等全身尿毒症临床表现，即提示急性肾功能衰竭者，我们予CRRT减轻脏器负荷。7例患者的TLS相关指标，均在加强干预后的2 d到1周内逐渐恢复至正常。1例按停药前剂量继续治疗，1例在再次降白细胞预处理后重新开始原方案，4例继续原方案逐步爬坡增加维奈克拉用量，1例因合并感染病情过重放弃治疗。

因此，维奈克拉联合去甲基化药物在急性白血病患者治疗过程中，可诱发以高钾、高磷、高尿酸、低钙、肾损害等为特征的TLS。尽管发生率不高，但也需要临床医师在用药前加强预防，尤其是在患者存在基础疾病病史、肿瘤负荷高、合并感染或出凝血异常时。临床医师应严格遵循维奈克拉的剂量爬坡给药原则，注意合并用药过程中维奈克拉的剂量调整，并在用药过程中密切监测血生化中TLS相关指标；一旦发生TLS，尽早干预处理。
